# Editorial: Chromosome segregation and aneuploidy in cancer

**DOI:** 10.3389/fcell.2023.1353851

**Published:** 2023-12-22

**Authors:** Oscar Molina, Jordi Camps

**Affiliations:** ^1^ Josep Carreras Leukemia Research Institute, Department of Biomedicine, School of Medicine, University of Barcelona, Barcelona, Spain; ^2^ Red Española de Terápias Avanzadas (TERAV), Instituto de Salud Carlos III, Barcelona, Spain; ^3^ August Pi i Sunyer Biomedical Research Institute (IDIBAPS), Barcelona, Spain; ^4^ Centro de Investigación Biomédica en Red en Enfermedades Hepáticas y Digestivas (CIBEREHD), Madrid, Spain; ^5^ Cell Biology and Medical Genetics Unit, Department of Cell Biology, Physiology and Immunology, Faculty of Medicine, Autonomous University of Barcelona (UAB), Bellaterra, Spain

**Keywords:** aneuploidy, whole-genome doubling (WGD), chromosomal instability (CIN), cancer, centromere (CEN)

The study of the cellular mechanisms involved in the faithfulness of chromosome segregation during cell division, the impact of chromosome missegregation and the resulting aneuploidy on cell physiology are areas of intense research. The functional association between these processes gained further interest when it became evident that despite the negative consequences of aneuploidy in the fitness of physiologically normal cells, it is the most common genomic abnormality in cancer cells, thus creating a paradox in terms of its contribution to tumorigenesis, the so-called “aneuploidy paradox.” This Research Topic explores the complex relationship between the causes and consequences of aneuploidy, as well as the contribution of abnormal chromosome numbers to cancer initiation and progression. Research teams at the forefront of Chromosome and Cancer Biology from different countries brought their unique perspective on these questions and contributed with excellent review articles to this Research Topic collection.

Van den Berg and Jansen review the role of SUMOylation in centromere stability and function, which is necessary for faithful chromosome segregation. Centromeres are the site of assembly of the kinetochore, a proteinaceous structure that directs chromosome segregation during cell division. Active centromeres are characterized by the presence of nucleosomes containing the centromere-specific histone H3 CENP-A and a specific chromatin environment that resembles that of active genes. Thus, providing evidence for a fine balance between different histone post-translational modifications at centromere sites and kinetochore function. The authors highlight the important role of SUMO modification on maintaining CENP-A chromatin stability, ensuring proper kinetochore strength at the centromere while preventing ectopic centromere formation.

Sanz-Gómez et al. explore the complex relationship between whole-genome doubling (WGD) and the resulting polyploidy in cancer. In this review, authors describe the cellular mechanisms leading to WGD in normal cells and in the tumoral context, exploring the effects of WGD on the prognosis of different types of cancers. Since polyploidy is associated with detrimental effects to cell fitness, authors focussed this review on the cellular mechanisms leading to cellular adaptation to tetraploidy and the resulting high levels of chromosomal instability, which ultimately trigger cancer.

In their review, Cimini and Bloomfield describe the recent findings aiming at understanding how extra centrosomes acquired as a result of whole genome duplication events result in aneuploidy and chromosome instability. Accumulating evidence suggest that following a whole genome duplication, extra centrosomes undergo a process of dynamic evolution to reach a centrosome homeostasis, which usually involves centrosome loss in order to prevent multipolar cell divisions. The authors also review the functional consequences of whole genome duplication and extra centrosomes, giving the fact that the presence of extra centrosomes has been shown to be causative of invasive phenotypes, a canonical feature of cancer cells.

Ragusa and Vagnarelli review the contribution of different histone variants to chromosome segregation errors leading to aneuploidy in cancer. Recent data revealed the presence of mutations, aberrant expression patterns and/or post-translational modifications of a wide variety of histone variants in several cancers, which are associated with chromosome missegregation events. In this systematic review article, authors compiled a database of histone gene alterations linked to aneuploidy in cancers of the TCGA project.

Overall, this Research Topic brings to the forefront the complex relationship between chromosome segregation defects and the resulting aneuploidy on cancer initiation and progression. Importantly, it is evident that the cellular mechanisms leading to tolerance to aneuploid karyotypes generated by chromosomal instability can be exploited therapeutically to improve the clinical outcome in cancer patients.

